# Get to Know Your Neighbors: Characterization of Close *Bacillus anthracis* Isolates and Toxin Profile Diversity in the *Bacillus cereus* Group

**DOI:** 10.3390/microorganisms11112721

**Published:** 2023-11-07

**Authors:** Mehdi Abdelli, Charlotte Falaise, Valérie Morineaux-Hilaire, Amélie Cumont, Laurent Taysse, Françoise Raynaud, Vincent Ramisse

**Affiliations:** 1 DGA CBRN Defence Center, Biology Division, French Ministry of the Armed Forces, 91710 Vert-le-Petit, France; mehdi.abdelli@intradef.gouv.fr (M.A.); valerie.morineaux-hilaire@intradef.gouv.fr (V.M.-H.); amelie.cumont@intradef.gouv.fr (A.C.); laurent.taysse@intradef.gouv.fr (L.T.); francoise.raynaud@intradef.gouv.fr (F.R.); 2 Institute for Integrative Biology of the Cell (I2BC), CNRS, Université Paris-Saclay, 91190 Gif-sur-Yvette, France

**Keywords:** *Bacillus anthracis*, *Bacillus cereus s.l.*, whole genome sequencing (WGS), SNP phylogeny, toxin genes, virulence factors, cereulide, MALDI-TOF MS, biovar Thuringiensis, biovar Emeticus

## Abstract

Unexpected atypical isolates of *Bacillus cereus s.l.* occasionally challenge conventional microbiology and even the most advanced techniques for anthrax detection. For anticipating and gaining trust, 65 isolates of *Bacillus cereus s.l.* of diverse origin were sequenced and characterized. The BTyper3 tool was used for assignation to genomospecies *B. mosaicus* (34), *B. cereus s.s* (29) and *B. toyonensis* (2), as well as virulence factors and toxin profiling. None of them carried any capsule or anthrax-toxin genes. All harbored the non-hemolytic toxin *nheABC* and sphygomyelinase *spH* genes, whereas 41 (63%), 30 (46%), 11 (17%) and 6 (9%) isolates harbored *cytK-2*, *hblABCD*, *cesABCD* and at least one insecticidal toxin gene, respectively. Matrix-assisted laser desorption ionization-time of flight mass spectrometry confirmed the production of cereulide (*ces* genes). Phylogeny inferred from single-nucleotide polymorphisms positioned isolates relative to the *B. anthracis* lineage. One isolate (BC38B) was of particular interest as it appeared to be the closest *B. anthracis* neighbor described so far. It harbored a large plasmid similar to other previously described *B. cereus s.l.* megaplasmids and at a lower extent to pXO1. Whereas bacterial collection is enriched, these high-quality public genetic data offer additional knowledge for better risk assessment using future NGS-based technologies of detection.

## 1. Introduction

Anthrax, a bacterial zoonosis transmissible to humans, is feared as a biological weapon or bioterrorism agent. The causative agent, *Bacillus anthracis*, has been listed by the Federal Select Agent Program as having the potential to pose a serious threat to public health [1], thus justifying its detection as confidently as possible even in the most challenging situations, such as complex or degraded samples. As major virulence factors, the tripartite anthrax-toxin genes *cya*, *lef* and *pag*, located on plasmid pXO1 (182 kb), and poly-γ-D glutamic acid capsule genes *capABCDE* on pXO2 (95kb) are common targets for detection [2,3]. With rare exceptions, confirmation of the identity of *B. anthracis* and differentiation from other *Bacillus* species is easy with traditional cultivation techniques for a trained eye [4]. Generic broad-spectrum techniques covering multiple pathogens are increasingly desirable, although significant developments have been accomplished, for example, using mass spectrometry (MS) and nanopore sequencing [5,6,7]. Thus, it is worthwhile to expand the sampling of strains likely to weaken all these techniques and to assess new strains as opportunities arise.

In addition to its clonal population structure, *B. anthracis* is part of a larger group of species with close phylogeny referred to as the *Bacillus cereus* group or *B. cereus* *sensu lato* (*B. cereus s.l.*). The description of at least 20 species over the last two decades, accompanied by the expansion of whole genome sequencing (WGS), has led to changes from the traditional “legacy” nomenclature [8,9]. A combined genomospecies–subspecies–biovar nomenclature framework was recently proposed [10]. Note that examples of misinterpretation of *B. cereus* group WGS results call for caution from those who are transitioning to WGS for *B. cereus* group strain characterization [11].

Some *B. cereus s.l.* isolates can produce parasporal crystal proteins (Cry, Cyt) with pesticidal properties used worldwide for pest-control in agriculture. Such strains, historically gathered under the name *B. thuringiensis*, coincide with diverse species of the *B. cereus* group and are now referred to as biovar Thuringiensis with the novel nomenclature framework [10]. Updated nomenclature of crystal protein genes is available [12]. Some other *B. cereus s.l.* isolates are responsible for emetic and/or diarrheal foodborne illness or intoxication [13,14]. Briefly, the heat-stable emetic toxin cereulide encoding gene cluster *cesABCD* is located on a large plasmid [15,16]. The term biovar Emeticus is used in the novel nomenclature framework to describe these cereulide producers [10]. The second syndrome is caused by three thermolabile enterotoxins, hemolysin BL (HBL), non-hemolytic enterotoxin (NHE) and cytotoxin K (CytK), chromosomally encoded by the operons *nheABC* and *hblCDAB* and the genes *cytK-1* or *cytK-2*, respectively. Massive horizontal gene transfer has shaped the evolution of the *B. cereus s.l.* enterotoxin operons *hbl*, *nhe* and *cytK* [17].

*B. cereus s.l.* lineages have close evolutionary relationships and their possible ecological niches and lifestyles are still under elucidation [18,19]. Naturally occurring isolates belonging to the *B. cereus* group with anthrax-toxin genes are able to cause fatalities even in humans [20] (referred to as biovar Anthracis isolates [10]). Some *B. cereus s.l.* causing anthrax produce an exo-polysaccharide capsule encoded by the plasmidic operon *bpsABCDEFGHX* [20,21,22] alternatively to the regular poly-γ-D glutamic acid capsule encoded by pXO2-*cap* genes [23,24]. *B. cereus s.l.* G9241 elaborates a hyaluronic acid capsule via plasmidic *hasABC* genes, unlike *B. anthracis* which has a mutation preventing the translation of *hasA* [25]. Recently, a retrospective screening of an anthrax-like disease induced by a strain of *Bacillus tropicus* from Chinese turtles in Taiwan reinforced the idea that the host range and geographic distribution of atypical *B. cereus s.l.* are by far underestimated [26].

Virulence determinants of anthrax and anthrax-like isolates are a tiny part of their genome carried, in the main, by mobile elements. False-positive detection issues are critical in complex environments, particularly because promising technologies are rather generic and increasingly sensitive. For instance, a weak metagenomics detection signal of anthrax in air samples of the New York subway wrongly suggested that the pathogen was present in trace amounts in the normal urban microbiome [27]. The study design did not include sample cultivation, thus hampering any chance to trace back to the probable source of such signal. Similarly, metagenome sequencing of an anthrax-negative soil sample using CRISPR-Cas-based detection showed thousands of reads mapped on strain *B. anthracis* Ames Ancestor. No phylogenetic localization was made, although this would have shed light on the nature of this signal [28].

As a result, confident discriminative detection between harmless or any anthrax-causing isolates is relevant for both public health and biodefence. Trust will depend on our extended sampling efforts within the *B. cereus s.l.* lineages. Even if rare or hypothetically underestimated, such atypical lineages should not be ignored.

Aiming to gain an increased representativeness of *B. cereus* group lineages that may improve our reference collection and subsequent reference databases, we seized the opportunity to characterize a collection of *B. cereus s.l.* isolated from clinical specimens, food including dairy products, and water during the 2007–2015 period in Slovenia. Identification using traditional bacteriology techniques preceded WGS-based species classification and toxin profiling with BTyper3 [29], whereas matrix-assisted laser desorption ionization-time of flight mass spectrometry (MALDI-TOF MS) provided the production status of cereulide. Phylogeny inferred from whole genome single-nucleotide polymorphism (SNP) analysis placed them in a larger context within the *B. cereus* group in order to highlight those closer to the *B. anthracis* lineage and to improve our exclusivity panel.

## 2. Materials and Methods

### 2.1. Strains and Cultivation

Strains listed in Supplementary Table S1 were handled in a biosafety level 2 (BSL-2) laboratory. Sixty-five *Bacillus* isolates were kindly provided by Institute of Microbiology and Immunology, Faculty of Medicine, University of Ljubljana (Ljubljana, Slovenia) and were collected between 2007 and 2015 from multiple isolation sources: thirty isolates from clinical specimens, twenty-one from dairy products, nine from foods and five from water. Some isolates were previously investigated for their antimicrobial susceptibility and their pulsotype diversity [30,31]. Strains were purity-checked upon receipt on tryptic soy agar plates supplemented with 5% sheep blood (TSS-agar; BioMérieux, Marcy l’Etoile, France). The hemolytic activity was assessed on TSS-agar and the phospholipase activity was determined on the selective *B. cereus* group BACARA agar (BioMérieux, Marcy l’Etoile, France). All cultures were incubated for 18 h ± 2 h at 37 °C. Gram staining and microscopic observations were conducted using classical procedures.

### 2.2. MALDI-TOF Mass Spectrometry

Prior to matrix-assisted laser desorption/ionization-time of flight mass spectrometry (MALDI-TOF MS) analysis, the isolates were cultivated on TSS agar for 18 ± 2 h at 37 °C. A freshly grown colony sample was picked with a 1 µL sterile loop and a thin film was smeared and left to dry on a 96-polished steel target plate (Bruker Daltonik GmbH, Bremen, Germany). Samples were overlaid with 1 µL of the matrix α-cyano-4-hydroxycinnamic acid (HCCA, Bruker Daltonik GmbH, Bremen, Germany) prepared following the instructions for use and with a final concentration of 10 mg/mL. The matrix was left to crystallize for 10 min at room temperature before mass spectra acquisition of the samples with an MALDI Biotyper^®^ Sirius System (Bruker Daltonik GmbH, Bremen, Germany). The data were processed automatically using MBT Compass 4.1.100 software (Bruker Daltonik GmbH, Bremen, Germany) with default parameters (MBT_AutoX AutoXecute method and MBT_Process processing method). The instrument was calibrated in the range of 3637.8–16,953.3 Da using Bruker Bacterial Test Standard (Bruker Daltonik GmbH, Bremen, Germany). The mass spectra obtained from the isolates were compared with those of known microbial isolates of the commercial libraries provided by Bruker Daltonik, including the MBT Compass Library BDAL (Revision H, 2021) and the MBT Security Related Library 1.0 (SR). For the *B. cereus* group, the BDAL library includes strains of the following species: *B. cereus sensu stricto* (×4), *B. thuringiensis* (×1), *B. mycoides* (×1), *B. pseudomycoides* (×1), *B. weihenstephanensis* (×1) and *B. cytotoxicus* (×4), and the SR library includes *B. anthracis* (×23). The degree of correspondence between the test spectrum and the reference spectra in the database is expressed with log(score) values between 0 and 3.0, with a log(score) ≥ 2.0 indicating that identification could be reliable at the species level of the organism. Each strain was spotted on at least two different spots and the spectrum with the best log(score) was taken into account.

### 2.3. Genomic DNA Extraction

Bacterial biomass was collected from isolation streaks on a TSA agar plate (BioMérieux SA, Marcy l’Etoile, France) incubated 18 ± 2 h at 37 °C. The biomass was transferred in 200 µL of sterile water and bead-grinded for 45 s at 6000 rpm with a Precellys Evolution homogenizer (Bertin Technologies SAS, Montigny-le-Bretonneux, France). Genomic DNA was extracted with a DNeasy^®^ Blood & Tissue kit (Qiagen, Hilden, Germany) following the manufacturer’s recommendations. Eluted DNA solution was sterilized via centrifugation (4 min, 12,000× *g* rpm) on 0.2 µm filter microtubes (Merck KGaA, Darmstadt, Germany). DNA quality and concentration were estimated with a spectrophotometer/fluorometer DS-11 Series (DeNovix, Wilmington, DL, USA) and using a Qubit dsDNA HS Assay kit (Invitrogen, Thermo Fisher Scientific, Waltham, MA, USA).

### 2.4. Illumina Sequencing, De Novo Assemblies and Ames Ancestor-Reference-Based Assemblies

A paired-end library was constructed using Nextera XT DNA library Prep Kit and sequenced on an Illumina MiSeq platform. Low-quality bases were removed using *Trimmomatic* v0.39 [32] and reads were de novo assembled into contigs using SPAdes v3.13.0 [33] (default parameters). For reference-based assembly, reads were mapped against the chromosome sequence of *B. anthracis* Ames Ancestor A2084 (GCF_000008445.1) using BioNumerics 8.1.1 (BioMérieux, Applied Maths, Sint-Martens-Latem, Belgium) with default parameters and the following options: “perform gapped alignments” (enabled).

### 2.5. Nanopore Sequencing and De Novo Hybrid Assembly

Genomic DNA of BC38B strain was sequenced for 24 h on a MinION system with a FLO-MIN106 flow cell (R9 version) using a ligation sequencing kit SQK-LSK109 (ONT). Reads were basecalled and demultiplexed using Guppy v6.0.7 (super-accurate mode) [34]. Reads were filtered with a q-score threshold of 10 during guppy basecalling. Adapters were trimmed from the reads using Porechop v0.2.4 [35]. Hybrid (Illumina and MinION reads) de novo assembly was performed using SPAdes v3.13.0. Default parameters were used for these software.

### 2.6. Nucleotide Sequence Accession Numbers 

The assemblies were deposited in DDBJ/ENA/GenBank as BioProjects PRJNA945829 and PRJNA891199.

### 2.7. Public Genomes

In order to place the strains of this study in a larger phylogenetic context, RefSeq genome assemblies of the *B. cereus* group were downloaded at NCBI (last update: 22 January 2023). Because of different assembly levels (complete, scaffold, contig), data were converted to simulated reads using an in-house script (Python v3.6.2) and then mapped to Ames Ancestor chromosome A2084 (GCF_000008445.1). The resulting alignment spanning set with identical length served for genomic SNP analysis as detailed later in the text. Establishing phylogeny of relatives to anthrax justifies Ames Ancestor as appropriate reference and remains reliable since *B. cereus* group members have close phylogeny. The selection of public genomes included the following: (1) a “*B. cereus* group” panel with 25 public genomes spanning over the genomospecies *B. mosaicus*, *B. cereus s.s.*, *B. luti* and *B. toyonenis* as defined by Carroll et al. [10] (reference genomes of the different species proposed by the NCBI and/or by Carroll et al. [8,10]); (2) a “anthrax toxin gene-harboring genomes” panel with 12 genomes that do not belong to the clonal *B. anthracis* lineage, as defined by Carroll et al. [36]; and (3) a “*B. anthracis*” panel with 24 public genomes representing major lineages and sublineages of *B. anthracis* as described previously [37]. The contents of these panels are summarized in Supplementary Table S2. 

In addition, in order to highlight strains from this study that are the closest to *B. anthracis*, all the “*Bacillus cereus* group” assemblies were downloaded from the NCBI database (3950 genomes classified into 24 different species, January 2023). The reconstructed genomes exhibiting at least 70% of nucleotide sequence identity with Ames Ancestor were retained (*n* = 178) as the overall population neighboring *B. anthracis* (Supplementary Table S3).

### 2.8. Whole Genome SNPs and Population Clustering Analysis

Whole genome SNP analysis with BioNumerics 8.1.1 used the option “Strict SNP filtering (Closed SNP set)” with default parameters (12 bp inter-SNP), and the detected SNPs were used for population clustering using the “Maximum parsimony tree” (MPT) calculation method with default parameters.

### 2.9. Genomospecies Assignment and Virulence Factor Detection 

An in silico characterization of the draft genomes was performed using BTyper3 tool [29]. It enabled an average-nucleotide-identity (ANI)-based genomospecies assignment, an ANI-based subspecies assignment, an eight-group adjusted *panC* group assignment, the identification of the sequence type (ST) and clonal complex, the screening of the main virulence factors within the *B. cereus* group and the detection of some pesticidal toxins. The BtToxin_Digger tool [38], including all referenced pesticidal toxins on the Bacterial Pesticidal Protein Resource Center database [12], was used to complete Btyper3 screening. Default parameters were used for these software.

In addition, all the draft genomes were uploaded to the Type Strain Genome Server (TYGS) [39,40,41], in user submission mode, to confirm with digital DNA/DNA hybridization values the closest reference species for each strain obtained by Btyper3 through pairwise genomic analysis.

### 2.10. Plasmid Analysis 

Due to its proximity with the *B. anthracis* cluster, an analysis of the strain BC38B was performed with a particular focus on its plasmid. The NCBI non redundant nucleotide database (last update: 22 April 2023) was used to search similar plasmid sequences in comparison to the BC38B plasmid. The DNA sequences of these closest plasmids were downloaded and compared with BLAST Ring Image Generator (BRIG) [42]. To identify minireplicons contained in the BC38B plasmid, a BLASTP analysis was performed against replication proteins previously described in the megaplasmids of the *B. cereus* group [43]. In order to potentially identify new types of minireplicons, the keywords “replication protein” or “Rep protein” or “Primase” were searched from the annotation file. TubZ protein sequences were also investigated with the same method. In addition, Ori-Finder 2022 (accessed on 23 April 2023) [44] was used to find the predicted origins of replication in the BC38B plasmid. 

### 2.11. MALDI-TOF Cereulide Production Detection

Cereulide toxin peaks were searched at 1175 *m*/*z* and 1191 *m*/*z* as defined by Ducrest et al. [45]. The production of cereulide was assessed for the 12 strains that clustered close to the emetic *B. cereus* strain AH187. It included 11 strains exhibiting the cereulide synthetase genes *cesABCD* (*cesABCD*+) and one lacking the cereulide gene cluster. The emetic strain AND1407 (*cesABCD*+) was used as a positive control for cereulide production and the *B. cereus s.s.* collection strain ATCC 14579 (*cesABCD*−) as a negative control. Sample preparation of cereulide was performed with the smear method, where a fresh colony sample is directly spotted onto the MALDI target plate and covered by 1 µL of the HCCA matrix (also used for the bacteria identification described above). Each strain was spotted in triplicates. Spectra acquisition was conducted with flexControl Analysis software (Bruker Daltonik GmbH, Bremen, Germany) version 3.4 (Build 207.20). The following parameters were set: random walk shots of partial sample with 100 shots at a raster spot (500 single spectra accumulation); sample rate and digitizer 0.5 GS/s. The smartbeam laser was set to a linear positive mode in the range of 700–6080 Da with a frequency of 200 Hz. Basic laser settings were high voltage: ion source 1, 10.00 kV; ion source 2, 9.03 kV; Lens, 2.99 kV; pulsed ion extraction set to 130 ns. The external calibration of the instrument was performed using low mass range Peptide Calibration Standard II (Bruker Daltonik GmbH, Bremen, Germany) prepared following the manufacturer’s recommendations, covering a mass range of 700–3500 Da. The mass spectra obtained were manually analyzed using flexAnalysis software (Bruker Daltonik GmbH, Bremen, Germany) version 3.4 (Build 79) and each spectrum was subjected to spectral preprocessing procedures: smoothing using the SavitzkyGolay algorithm, baseline subtraction using the TopHat algorithm and peak detection using the centroid algorithm with a signal to noise threshold set at 4.

## 3. Results

### 3.1. Strain Isolation and Microbiology

Isolates showed great phenotypical diversity with grayish circular colonies ranging in size from 0.1 cm to 1.3 cm; showing entire or irregular margins; with an elevation either flat, raised or umbonate; and a smooth/glistening or rough surface (Figure S1). Most strains expressed on TSS medium showed strong to weak β-hemolysis (hemolysis clearly extending the colony margin, Figure S1A,B,D, or slight hemolysis below colonies, Figure S1C), but also γ-hemolysis (null hemolysis, Figure S1E) and α-hemolysis (partial greenish hemolysis, Figure S1F). Isolations on TSS also allowed us to separate some strains that were mixed in the original samples (hereinafter referred to as SIBC*XX*A or SIBC*XX*B). It also appears that the subculturing of some strains led to phenotypical variations (Supplementary Table S1 summarizes dimorphic isolates; an example is illustrated in Figure S1E,F). All strains showed typical coral-colored colonies surrounded by an opacification halo on BACARA medium as a result of phospholipase activity. Microscopic observations showed Gram-positive rod-shaped and sporulating cells, isolated or in chains of variable length. None of the strains studied showed microscopic or macroscopic characteristics of *B. anthracis*.

### 3.2. Identification with MALDI-TOF MS 

All isolates were identified using MALDI-TOF MS as belonging to the *B. cereus* group with a log(score) ≥ 2.0. As both the BDAL and the SR Bruker libraries were used for the mass spectra comparison, false-positive “*B. anthracis*” identification results were obtained for 53% of the isolates, while the other 47% were identified as “*B. cereus*”. Supplementary Table S4 summarizes the identification results and scores obtained for each isolate.

### 3.3. Phylogenetic Relationships between B. cereus s.l. Isolates Based on wgSNP Analyses

**Figure 1:**
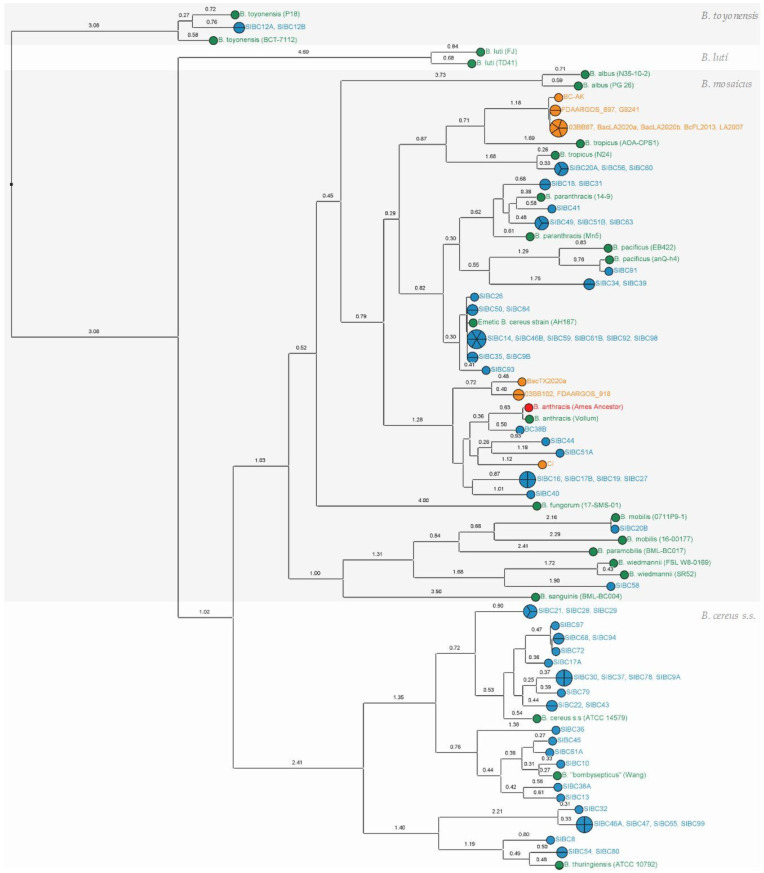
SNP phylogeny including “*B. cereus* group” panel with the public reference genomes of several *B. cereus s.l* species (in green), a panel with public genomes harboring anthrax-like genes (in orange) and strains from this study (in blue). Closely related *B. cereus s.l.* linages can be assigned to different genomospecies (*B. mosaicus*, *B. cereus s.s*, *B. luti* and *B. toyonensis*) as proposed by Carroll et al. [10]. Maximum parsimony tree built from 5929 SNP positions of 102 whole genomes mapped to the chromosomic genome of Ames Ancestor (in red, GCF_000008445.1). Numbers upon branches indicate the number of SNPs ×100. See Supplementary Table S2 for public strain details.

An SNP phylogeny, including all the strains from this study and a selection of public genomes (“*B. cereus* group” panel and “anthrax-toxin gene-harboring genomes” panel), was built and allowed for the taxonomic assignment of the studied isolates (Figure 1). Strains showed great genetic diversity across the three genomospecies *B. toyonensis* (×2), *B. mosaicus* (×34) and *B. cereus s.s* (×29), with isolates close to the reference strains of the species *B. anthracis*, *B. tropicus*, *B. pacificus*, *B. paranthracis*, *B. mobilis*, *B. wiedmannii*, *B. cereus s.s.*, *B. thuringiensis* and *B. toyonensis*.

### 3.4. Close B. anthracis Neighbors

**Figure 2:**
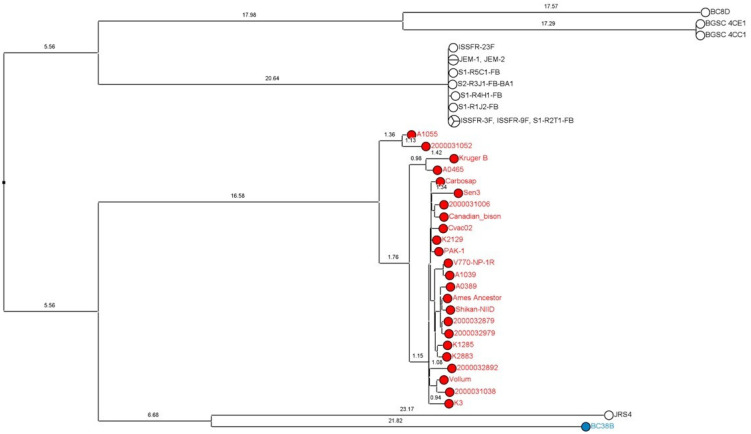
SNP phylogeny including the “*B. anthracis*” panel (in red) and the public genomes (Assembly) most closely related to the *B. anthracis* lineage (in white). The strain BC38B from this study (in blue) shares the fewest SNPs with *B. anthracis* in comparison with the other neighbors and appears to be the closest relative of the species. Maximum parsimony tree built from 13,826 SNP positions of 39 whole genomes mapped to the chromosomic genome of Ames Ancestor (GCF_000008445.1). Numbers upon branches indicate the number of SNPs ×100. See Supplementary Table S3 for strain details.

An SNP analysis and MPT clustering were conducted with the public genomes of *B. cereus s.l.* exhibiting ≥ 70% of nucleotide sequence identity with the Ames Ancestor chromosome but that are outside the *B. anthracis* lineage (*n* = 178). This analysis also included the “*B. anthracis*” panel and the eight isolates of this study close to the *B. anthracis* lineage (Supplementary Table S3). The closest *B. anthracis* neighbors were selected from the resulting SNP phylogeny and a second SNP analysis was conducted with the 14 closely related public genomes, the isolate BC38B from this study and the “*B. anthracis*” panel (Figure 2). It appeared from this SNP phylogeny that BC38B was the most closely related to the *B. anthracis* lineage in comparison with the public neighbors (4508 SNPs for BC38B vs. 4643 SNPs for the second-closest strain JRS4). 

### 3.5. Taxonomic Assignment and Virulence Factor Detection 

The taxonomic assignment for all strains and the screening for virulence factors are summarized in Table 1 (detailed results obtained with Btyper3, BtToxin_Digger and Type Strain Genome Server are available in Supplementary Table S5). 

Strains were assigned as follows: *B. toyonensis* (×2), *B. mosaicus* (×13), *B. mosaicus* biovar Thuringiensis (×6), *B. mosaicus* subsp. *cereus* (×4), *B. mosaicus* subsp. *cereus* biovar Emeticus (×11), *B. cereus s.s.* (×29). Virulence factors involved in the production of diarrheal toxins were found in all isolates. The non-hemolytic enterotoxin genes *nheABC* and the sphingomyelinase *spH* gene were detected in all genomes. The hemolysin BL genes *hblABCD* were harbored by 46% of the isolates and the diarrheal cytotoxin K variant 2 gene *cytk-2* by 63%. The gene coding for the diarrheal cytotoxin variant 1 was not detected in any of the isolates. Anthrax toxin genes *cya*, *lef* and *pagA* were not detected, nor were any of the capsule virulence factors. The cereulide synthetase genes *cesABCD* were harbored by all the isolates close to the reference emetic strain AH187, except for SIBC26. Six strains exhibited pesticidal toxin genes; these are detailed in Table 2. 

### 3.6. BC38B Plasmid Analysis

**Figure 3:**
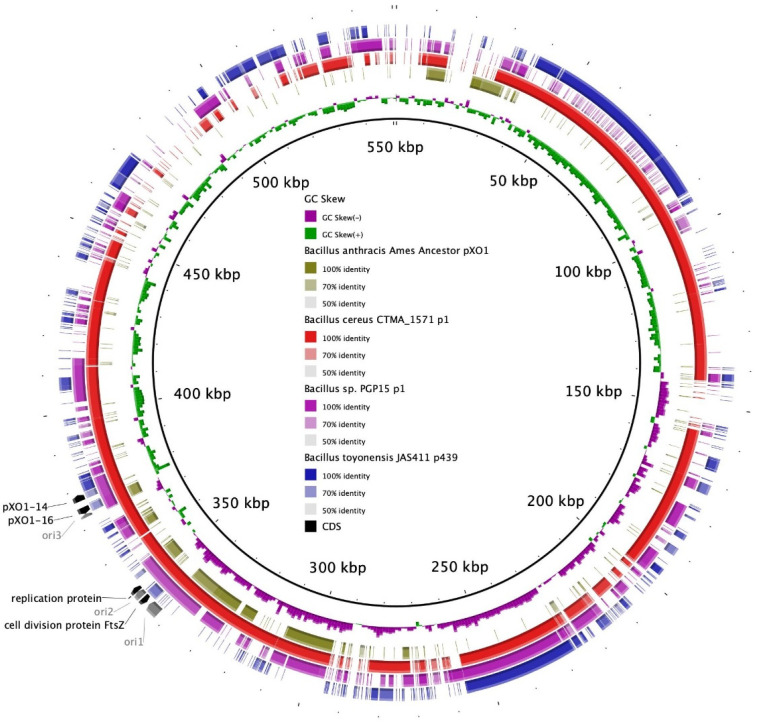
Comparison of BC38B plasmid to homologous plasmids (*B. cereus* strain CTMA_1571 plasmid p1, *B. toyonensis* strain JAS411 plasmid p439, and *Bacillus* sp. PGP15 plasmid p1) and *B. anthracis* Ames Ancestor pXO1 using Blast Ring Image Generator (BRIG). Rings from the center to the outermost: (1) scale marks; (2) GC skew; (3–6) sequence percentage identity to homologous plasmids; (7) minireplicon-associated protein coding genes (in black), origins of replication (in gray). The locations of shared regions between these plasmids relative to the BC38B plasmid are denoted in color in the figure. The color key corresponding to these designations is provided within the figure itself.

One megaplasmid of 551,060 kb was successfully reconstructed with hybrid de novo assembly. Minireplicon detection was performed on its sequence. A minireplicon represents the smallest region for plasmid replication and contains the origin of replication and genes encoding replication proteins. Three different origins of replication were determined on the sequence. Additionally, the plasmid exhibited the protein genes *pXO1-14/pXO1-16*-like (DNA-binding protein gene and replication initiator protein gene, respectively), which were first reported to support the replication of *B. anthracis* plasmid pXO1 [46]. A replication-relaxation protein coding gene and a cell division protein FtsZ coding gene were also detected. The first one is essential for plasmid DNA replication, while the second has a main role in cell division and duplication for plasmids [47]. A BLASTP analysis revealed that all these elements were widely distributed in the *B. cereus* group and could be defined as new minireplicons in the large plasmids of the *B. cereus* group. Their locations on the plasmid are shown in Figure 3 and correspond with regions of GC skew sign change, which coincide with the origin or terminus of replication [48].

### 3.7. Detection of Cereulide Production with MALDI-TOF MS

**Figure 4:**
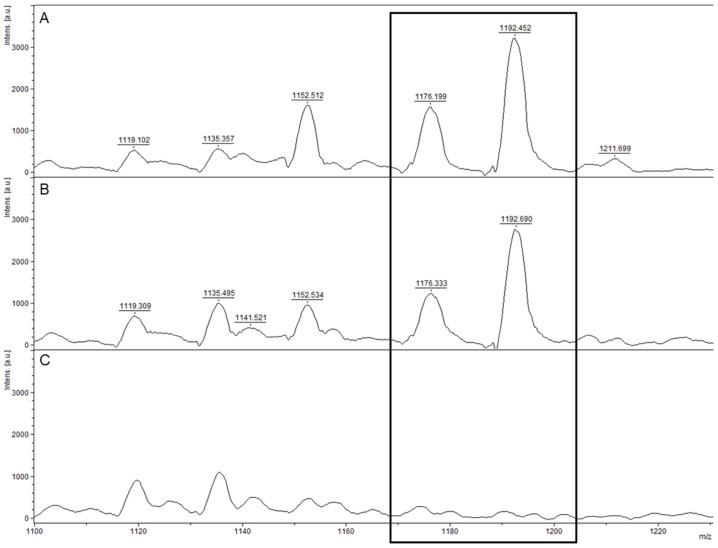
MALDI-TOF mass profiles of three isolates genetically close the emetic-type strain AH187 after wgSNP analysis. Mass spectrum corresponding to the colony smear of (**A**) the emetic collection strain AND1407, exhibiting the cereulide synthetase genes (*cesABCD*+) and showing the two cereulide characteristic peaks at 1176.1 and 1192.4 *m*/*z*; (**B**) the strain SIBC50 (*cesABCD*+), also exhibiting cereulide with peaks at 1176.3 and 1192.6 *m*/*z*; and (**C**) the strain SIBC26 (*cesABCD*−), lacking cereulide. Results shown are representative spectra of at least three independent experiments.

Eleven isolates found positive for *cesABCD* (Table 1) and phylogenetically grouped with the emetic *B. cereus* reference strain AH187 after wgSNP analysis (Figure 2) were verified for cereulide production using MALDI-TOF MS (SIBC14, SIBC46B, SIBC59, SIBC61B, SIBC92, SIBC98, SIBC50, SIBC84, SIBC35, SIBC9B and SIBC93). The isolate SIBC26 *cesABCD* negative (Table 1) within the same phylogroup (Figure 2) was also tested. The two characteristic cereulide peaks (1176 ± 1 *m*/*z* and 1192 ± 1 *m*/*z*) were detected for the 11 isolates that harbored the cereulide synthetase genes only (Figure 4).

## 4. Discussion

### 4.1. B. cereus s.l. Characterization

Isolates from this study showed notable phenotypic diversity with various colony morphologies among and within species (Table S1, Figure S1). Conventional microbiology techniques such as culturing remain an essential step for bacterial identification and various selective and/or chromogenic agar media have been developed for the isolation of *B. cereus s.l.* bacteria in complex matrices [49,50]. However, species of the *B. cereus* group cannot be distinguished based on morphological criteria, as characteristics used for taxonomic assignment (e.g., motility, hemolysis) vary within and among species [10,50]. For example, the lack of hemolytic activity on blood agar is a phenotypic characteristic used to discriminate *B. anthracis* from other *B. cereus s.l.*, but such features can vary depending on the *B. anthracis* strain and/or the blood origin [51]. Conversely, some non-*B. anthracis* strains lack hemolytic activity (e.g., isolate SIB79 from this study) and mislead the interpretation. The combination of conventional microbiology methods with advanced technologies such as molecular biology, biosensors or MALDI-TOF MS allows for a reliable identification [3].

Bacterial identification using MALDI-TOF MS is a recommendable first line technique for its speed and ease of use. All strains in this study were identified as members of the *B. cereus* group using MALDI-TOF MS with a high confidence score (log(score) ≥ 2.0). Moreover, it successfully identified cereulide-producing isolates. However, more than half were obviously misidentified as *B. anthracis* due to the incompleteness of the commercial library. The identification as either *B. cereus* or *B. anthracis* with MALDI-TOF did not reflect the genetic proximity of the isolate with these two species and it also appeared that different morphotypes of the same isolate could result in different species identifications (see Supplementary Table S3). Discrimination of closely related *B. cereus* group species using MALDI-TOF MS remains challenging, but the detection of species-specific biomarkers [52,53] and the expansion of the current commercial libraries with in-house reference libraries [6,54] could improve the statistical confidence of identification results and would avoid the occurrence of false-positive *B. anthracis* identification. 

WGS remains the gold standard for highly precise characterization, and tools such as BTyper3 [29] have been developed to easily assign a taxonomic affiliation and detect virulence factors in *B. cereus* group strains. In the present study, the identification of *B. anthracis* was ruled. Moreover, in silico ANI-based methods and digital DNA/DNA hybridization supported SNP phylogeny to reflect the diversity of the collection studied. 

### 4.2. Unnamed B. anthracis Neighbors

Eight isolates devoid of anthrax-virulence factors according to gene detection were nevertheless grouped proximally to the *B. anthracis* clade (Figure 1). Such genetic proximity is interesting, notably for biodefense, because part of the genome sequence information might constitute a potential source of false-positive signals of anthrax detection with metagenomics. In terms of nomenclature, as discussed by Carroll et al. [10], such isolates should not be referred to as *B. anthracis* to avoid incorrect assumptions of their anthrax-causing capabilities. For instance, these strains can be named after the genomospecies *B. mosaicus* which encompasses *B. anthracis* and other closely related species [8,10], but this taxonomic assignment does not underline the genetic proximity of these isolates with the *B. anthracis* species. It is very likely that the distinction of novel species for *B. anthracis*-close isolates will be described and will provide a better phylogenic characterization. Several strains have already been identified as closely related to *B. anthracis*, including strains that are colonizing the International Space Station [55] or the strain JRS4 isolated in the desert of Saudi Arabia [56,57]. Among the eight closest neighbors from this study, the isolate BC38B was especially interesting as it appeared to be the most closely related to the *B. anthracis* lineage in comparison with the other public isolates described so far (Figure 2).

### 4.3. Plasmid Analysis 

At least six types of minireplicons were discovered in the megaplasmids of the *B. cereus* group [43]. The presence of two or more minireplicons in a *B. cereus* group megaplasmid strongly suggests the integration of several smaller plasmids [43]. This type of integration event might have arisen for the BC38B plasmid. Indeed, each of the closest plasmid neighbors shared different regions with the BC38B plasmid (Figure 3). In particular, the strain CTMA_1571 plasmid p1 (Genbank accession number: CP053657.2) had a high homology with the BC38B plasmid and possessed the same minireplicon types. Both strains are close to the *B. anthracis* clade [58], although their sequence homology with the pXO1 plasmid is moderate. The BC38B plasmid also had regions of shared homology with plasmid p1 from *Bacillus* sp. PGP15 (isolated in soil from a rhizosphere in China; Genbank accession number: CP095875.1 [59]) and plasmid p439 from the *B. toyonensis* strain JAS411 (isolated in soil from a farmland in Poland; Genbank accession number: CP036114.1). These two strains belong to different clades of the *B. cereus* group, suggesting genetic exchange via horizontal transfer, which is a phenomenon that frequently occurred during the evolutionary history of the *B. cereus* group, even between phylogenetically distant strains [60].

### 4.4. Virulence Factor Detection and Toxin Profile Diversity 

The presence of toxin genes in *B. cereus s.l.* isolates was screened in a multitude of recent studies [8,61,62,63,64], and specific tools have been developed to ease their detection [29]. It appeared that the common distribution of virulence factors for the *B. cereus* group strains is the presence of approximately 85–100% *nhe* (*ABC*), 40–70% *hbl* (*CDA*), 40–70% *cytK-2*, very few *ces*+ and typically no *cytK-1*+ [13]. The screening of virulence factors conducted in the present work is totally consistent with this distribution, as *nhe* (*ABC*) were detected in 100% of the isolates, *hbl* (*CDA*) in 46%, *cytK-2* in 63%, the genes *ces* were detected in eleven isolates and none of the isolates were *cytK-1*+. The absence of *cytK-1* detection was expected as this enterotoxin is, for instance, only described in isolates of the species *B. cytotoxicus* [14]. It is also suggested that all *B. cereus* isolates can be categorized into seven different toxin profiles: A (*nhe*+, *hbl*+, *cytK*+), B (*nhe*+, *cytK*+, *ces*+), C (*nhe*+, *hbl*+), D (*nhe*+, *cytK*+), E (*nhe*+, *ces*+), F (*nhe*+) and G (*cytK*+) [65]. The isolates in this study therefore exhibit great toxin profile diversity with the presence of the categories A, C, D, E and F.

The observed toxin profile diversity within the panel of characterized strains highlighted that a nuanced, strain-specific approach to toxin analysis is essential as each isolate can display a unique set of challenges and implications. In a biodefence context, spotting the presence of specific toxins in strains that could be exploited for malevolent purposes is pivotal for the formulation of precise countermeasures and security protocols. For public health, the heterogeneity in toxin profiles directly influences the clinical presentation and outcome of infections. An understanding of the pathogenicity and virulence mechanisms associated with each strain would ensure timely and effective clinical interventions.

### 4.5. Biovars Anthracis, Emeticus and Thuringiensis 

Genomic determinants responsible for some phenotypes are plasmid-mediated (e.g., synthesis of anthrax toxin, bioinsecticide crystal proteins, emetic toxin synthetase proteins) and can be lost or gained within a species. The characterization of biovars in the “genomospecies/subsp./biovar” nomenclature framework proposed by Carroll et al. [8,10] allows one to highlight phenotypes of clinical and/or industrial importance.

The term “biovar Anthracis” is used for isolates that produce anthrax toxin (and/or possess anthrax-toxin encoded genes *cya*, *lef* and *pagA* [8]). *B. anthracis* biovar Anthracis (or *B.* Anthracis) has a long history as a life-threatening infectious agent to humans and animals worldwide [66] and has been extensively studied since the anthrax letter events in 2001 and the subsequent anthrax outbreaks [67,68]. The production of anthrax toxin has long been considered restricted to the *B. anthracis* species, but anthrax-causing strains have been characterized since 2006 outside the *B. anthracis* lineage in humans [8,23], great apes [24], in a kangaroo [8] and, recently, in a soft-shell turtle [26]. These *B. mosaicus* biovar Anthracis strains, previously referred to as “anthrax-*like*” strains, may exhibit different capsular composition [20] and are so far described as close *B. anthracis* neighbors or are related to the *B. tropicus* species [36] (see Figure 1 and Table 1). The isolation of such strains is rare and none were identified in the present study.

Apart from anthrax-causing strains, several other *B. cereus* group isolates are of great concern as they induce food intoxication and toxicoinfection resulting in vomiting, diarrhea and sometimes death [13,69]. The term “biovar Emeticus” is used for isolates known to produce cereulide (and/or possess the cereulide synthetase plasmid-encoded gene cluster *cesABCD* [8,70]). Emetic *B. cereus* strains (*B.* Emeticus) produce cereulide toxin during growth in food that causes vomiting, a progression referred to as emetic syndrome. Cereulide is a potent toxin, is heat- and acid-stable, and is responsible for an increasing number of foodborne poisonings that have gained public attention in recent years [14]. Cereulide toxin formation was thought to be restricted to a single evolutionary lineage of closely related strains [71], but cereulide-producing isolates have since been characterized across multiple lineages [70] (e.g., *B. mycoides* biovar Emeticus [72,73]). Detection methods for cereulide are improving and/or emerging [74] and the presence of the toxin can be certified using MALDI-TOF MS with rapidity and sensitivity from a colony smear [45,75]. In the present study, the gene cluster *cesABCD* was detected in 11 isolates and the production of cereulide was confirmed for each of them using MALDI-TOF MS (see Table 1 and Figure 4). All these strains were genetically close to the emetic reference strain AH187 (see Figure 1) and no other biovar Emeticus strain was detected in other lineages. 

The term “biovar Thuringiensis” is used for isolates that produce one or more *Bt* toxins (and/or possess *Bt* toxin-encoding genes [8]). *Bt* toxins are used as biopesticides in organic agriculture and are considered harmless to humans, although there is a growing concern that residues in food may occasionally cause diarrheal illness [76]. Indeed, diagnostic laboratories generally do not distinguish between *B. cereus s.s.* and *B.* Thuringiensis, potentially leading to misattributed cases of foodborne illnesses. This lack of distinction might result in underestimations of the disease impact associated with *B.* Thuringiensis [16]. The nomenclature of *Bt* toxin is complex and regularly subject to change as new genes involved in *Bt* toxin formation and new proteins with insecticidal properties are frequently discovered [77]. The production of *Bt* toxins is not restricted to isolates genetically close to the *B. thuringiensis* species and insecticidal activities have, for example, been described in strains close to the species *B. toyonensis* (*B. toyonensis* biovar Thuringiensis [78]) and *B. wiedmannii* (*B. mosaicus* biovar Thuringiensis [79,80]). In the present study, *Bt* toxin-encoding genes were detected in six isolates (see Table 1). These *B. mosaicus* biovar Thuringiensis (or *B.* Thuringiensis) strains were genetically close to the *B. paranthracis* or to the *B. anthracis* reference strains. None of the isolates genetically close to the *B. thuringiensis* species reference strain ATCC10792 possessed *Bt* toxin-encoding genes.

## 5. Conclusions

In this report, we have presented a detailed characterization of 65 strains of *B. cereus s.l.*, completely sequenced and identified as *B. mosaicus*, *B. cereus s.s.* and *B. toyonensis* using bacterial genomic taxonomy. Whatever their origin, clinical, food or water, the identification of *B. anthracis* has been excluded. All strains carried non-hemolytic enterotoxin genes, including those carrying crystal protein genes (biovar Thuringiensis). Such strains, although lacking BL hemolysin, are not completely devoid of diarrheal pathogenic potential, and this raises the issue to what extent biopesticides are free of any risk, especially because additional acquisition via horizontal gene transfer might not be inconceivable. In this way, our work contributed to the surveillance of circulating isolates and any contributions in this direction will be worthwhile. We also described the strain BC38B, carrying a megaplasmid, that was positioned phylogenetically closer to *B. anthracis* than those already described, which constitutes an encouraging additional step towards establishing the age of the *B. anthracis* species or even of the most recent common ancestor of already identified lineages. Overall, this work constitutes a valuable source of information and biological resources intended to better take into account the biological risk, whether in service of public health or biodefense.

## Figures and Tables

**Table 1:** Screening of major toxin-virulence genes among strains of the present study and among the public strains of the “*B. cereus* group” panel (in green) and “anthrax-toxin gene-harboring genomes” panel (in orange) using the BTyper3 tool. “+”: presence; “−“: absence; “(+)”: partially detected (i.e., not all the genes of the cluster were detected). The taxonomic assignment was based on in silico DNA/DNA hybridization and confirmed with Btyper3 tool.

Strain id.	Virulence Factors											Closest Reference Species	Taxonomic Assignment by BTyper3
	*nheABC*	*hblABCD*	*cesABCD*	*cytK-1*	*cytK-2*	*spH*	*Bt* toxins *	*cya, lef, pagA*	*capABCDE*	*hasABC*	*bpsABCDEFGHX*		
*B. toyonensis* (BCT-7112), *B. toyonensis* (P18)	+	+	−	−	−	+	−	−	−	−	(+)	*B. toyonensis*	*B. toyonensis*
SIBC12A, SIBC12B	+	+	−	−	−	+	−	−	−	−	(+)	*B. toyonensis*	*B. toyonensis*
*B. luti* (TD41), *B. luti* (FJ)	+	−	−	−	−	+	−	−	−	−	(+)	*B. luti*	*B. luti*
*B. paramobilis* (BML-BC017)	+	+	−	−	−	+	−	−	−	−	(+)	*B. paramobilis*	*B. mosaicus*
*B. mobilis* (16-00177), *B. mobilis* (0711P9-1)	+	−	−	−	−	+	−	−	−	−	(+)	*B. mobilis*	*B. mosaicus*
SIBC20B	+	−	−	−	−	+	−	−	−	−	(+)	*B. mobilis*	*B. mosaicus*
*B. wiedmannii* (FSL W8-0169)	+	+	−	−	+	+	−	−	−	−	(+)	*B. wiedmannii*	*B. mosaicus*
*B. wiedmannii* (SR52)	+	+	−	−	−	+	−	−	−	−	(+)	*B. wiedmannii*	*B. mosaicus*
SIBC58	+	+	−	−	−	+	−	−	−	−	(+)	*B. wiedmannii*	*B. mosaicus*
*B. sanguinis* (BML-BC004)	+	−	−	−	−	+	−	−	−	−	(+)	*B. sanguinis*	*B. mosaicus*
*B. tropicus* (N24)	+	−	−	−	+	+	−	−	−	−	(+)	*B. tropicus*	*B. mosaicus*
SIBC56, SIBC60, SIBC20A	+	−	−	−	+	+	−	−	−	−	(+)	*B. tropicus*	*B. mosaicus*
G9241, FDAARGOS_897, 03BB87, LA2007, BacLA2020a, BacLA2020b	+	+	−	−	+	+	−	+	−	+	+	*B. tropicus*	*B. mosaicus * biovar Anthracis ^(1)^
BcFL2013	+	+	−	−	+	+	−	+	−	+	(+)	*B. tropicus*	*B. mosaicus * biovar Anthracis ^(1)^
BC-AK	+	+	−	−	+	+	−	(+)	+	+	(+)	*B. tropicus*	*B. mosaicus * biovar Anthracis ^(1)^
*B. tropicus* (AOA-CPS1)	+	+	−	−	+	+	−	−	−	−	(+)	*B. tropicus*	*B. mosaicus*
*B. pacificus* (EB422)	+	−	−	−	+	+	−	−	−	−	(+)	*B. pacificus*	*B. mosaicus*
*B. pacificus* (anQ-h4)	+	−	−	−	−	+	−	−	−	−	(+)	*B. pacificus*	*B. mosaicus*
SIBC91	+	−	−	−	−	+	−	−	−	−	(+)	*B. pacificus*	*B. mosaicus*
SIBC34, SIBC39	+	+	−	−	−	+	+	−	−	−	(+)	*B. paranthracis*	*B. mosaicus* biovar Thuringiensis ^(3)^
*B. paranthracis* (Mn5)	+	−	−	−	+	+	−	−	−	−	(+)	*B. paranthracis*	*B. mosaicus * subps. *cereus* ^(4)^
SIBC18, SIBC31	+	−	−	−	−	+	−	−	−	−	(+)	*B. paranthracis*	*B. mosaicus* subsp. *cereus* ^(4)^
SIBC49, SIBC63, SIBC51B	+	−	−	−	+	+	+	−	−	−	(+)	*B. paranthracis*	*B. mosaicus* biovar Thuringiensis ^(3)^
*B. paranthracis* (14-9)	+	−	−	−	−	+	−	−	−	−	(+)	*B. paranthracis*	*B. mosaicus *subps. *cereus* ^(4)^
SIBC41	+	−	−	−	−	+	−	−	−	−	(+)	*B. paranthracis*	*B. mosaicus* subsp. *cereus* ^(4)^
SIBC93	+	−	+	−	−	+	−	−	−	−	(+)	*B. paranthracis* ^(5)^	*B. mosaicus* subsp. *cereus* biovar Emeticus ^(6)^
Emetic *B. cereus* strain (AH187)	+	−	+	−	−	+	−	−	−	−	(+)	*B. paranthracis* ^(5)^	*B. mosaicus * subsp. *cereus* biovar Emeticus ^(6)^
SIBC14, SIBC46B, SIBC59, SIBC61B, SIBC92, SIBC98, SIBC50, SIBC84, SIBC35, SIBC9B	+	−	+	−	−	+	−	−	−	−	(+)	*B. paranthracis* ^(5)^	*B. mosaicus* subsp. *cereus* biovar Emeticus ^(6)^
SIBC26	+	−	−	−	−	+	−	−	−	−	(+)	*B. paranthracis* ^(5)^	*B. mosaicus* subsp. *cereus* ^(4)^
03BB102, FDAARGOS_918, CI	+	−	−	−	−	+	−	+	+	+	(+)	*B. anthracis*	*B. mosaicus * biovar Anthracis ^(1)^
BacTX2020a	+	−	−	−	−	+	−	+	−	+	(+)	*B. anthracis*	*B. mosaicus * biovar Anthracis ^(1)^
*B. anthracis* (Ames Ancestor), *B. anthracis* (Vollum)	+	−	−	−	−	+	−	+	+	+	(+)	*B. anthracis*	*B. mosaicus * subsp. *anthracis* biovar Anthracis ^(1,2)^
BC38B	+	−	−	−	−	+	−	−	−	−	(+)	*B. anthracis*	*B. mosaicus*
SIBC44, SIBC51A, SIBC16, SIBC19, SIBC27, SIBC17B	+	−	−	−	+	+	−	−	−	−	(+)	*B. anthracis*	*B. mosaicus*
SIBC40	+	−	−	−	−	+	+	−	−	−	(+)	*B. anthracis*	*B. mosaicus* biovar Thuringiensis ^(3)^
*B. albus* (N35-10-2), *B. albus* (PG 26)	+	+	−	−	−	+	−	−	−	−	(+)	*B. albus*	*B. mosaicus*
*B. fungorum* (17-SMS-01)	+	(+)	−	−	−	+	−	−	−	−	(+)	*B. fungorum*	*B. mosaicus*
*B. cereus s.s.* (ATCC 14579)	+	+	−	−	+	+	−	−	−	−	(+)	*B. cereus s.s.*	*B. cereus s.s.*
SIBC21, SIBC28, SIBC29, SIBC22, SIBC43, SIBC30, SIBC37, SIBC78, SIBC9A, SIBC79, SIBC68, SIBC94, SIBC72, SIBC97, SIBC17A	+	+	−	−	+	+	−	−	−	−	(+)	*B. cereus s.s.*	*B. cereus s.s.*
*B. “bombysepticus”* (Wang)	+	+	−	−	+	+	−	−	−	−	(+)	*B. cereus s.s.*	*B. cereus s.s.*
SIBC13, SIBC38A, SIBC10, SIBC45, SIBC61A, SIBC36	+	+	−	−	+	+	−	−	−	−	(+)	*B. cereus s.s.*	*B. cereus s.s.*
*B. thuringiensis* (ATCC 10792)	+	+	−	−	+	+	+	−	−	−	(+)	*B. thuringiensis*	*B. cereus s.s. * biovar Thuringiensis ^(3)^
SIBC8, SIBC54, SIBC80, SIBC32	+	+	−	−	+	+	−	−	−	−	(+)	*B. thuringiensis*	*B. cereus s.s.*
SIBC47	+	−	−	−	+	+	−	−	−	−	(+)	*B. thuringiensis*	*B. cereus s.s.*
SIBC65	+	−	−	−	+	+	−	−	−	−	(+)	*B. thuringiensis*	*B. cereus s.s.*
SIBC99	+	−	−	−	+	+	−	−	−	−	(+)	*B. thuringiensis*	*B. cereus s.s.*
SIBC46A	+	−	−	−	+	+	−	−	−	−	(+)	*B. thuringiensis*	*B. cereus s.s.*

* Insecticidal toxins, ^(1)^ *B. Anthracis*, ^(2)^ *B. anthracis* biovar Anthracis, ^(3)^ *B. Thuringiensis*, ^(4)^ *B. cereus*, ^(5)^ Emetic *B. cereus* reference strain, ^(6)^ *B. Emeticus*.

**Table 2:** List of pesticidal toxins detected among strains of the present study using BtToxin_Digger.

Strain ID	Pesticidal Toxins
SIBC34	Cry28Aa2, Mpp64Aa1, Vpb4Aa1, Spp1Aa1
SIBC39	Cry28Aa2, Mpp64Aa1, Vpb4Aa1, Spp1Aa1
SIBC40	Cry1Ie5, Spp1Aa1
SIBC49	Cry13Aa2, Cry41Ba2, Spp1Aa1
SIBC51B	Cry13Aa2, Cry41Ba2, Spp1Aa1
SIBC63	Cry13Aa2, Cry41Ba2, Spp1Aa1

## Data Availability

The assemblies have been deposited in DDBJ/ENA/GenBank under the accession numbers referenced in Supplementary Table S1.

## Supplementary Materials

The following supporting information can be downloaded at https://www.mdpi.com/article/10.3390/microorganisms11112721/s1, Figure S1: Phenotypic diversity of several *B. cereus s.l* strains on tryptic soy agar + 5% sheep blood after an 18 h incubation at 37 °C. Colony pictures of (A) SIBC91 (*B. pacificus* species); (B) SIBC38A (*B. cereus s.s.*); (C) SIBC17A (*B. cereus s.s.*); (D) SIBC12A (*B. toyonensis*); and (E,F) two morphotypes of SIBC41 (*B. paranthracis*). Scale bars 0.5 cm; Table S1: Summary of the characteristics of the studied strains and their corresponding assemblies; Table S2: Composition of the three public genome datasets used in this study: (1) the “*B. cereus* group” panel with reference/representative strains of several *B. cereus s.l.* species according to the NCBI database and/or according to Carroll et al. [8]; (2) the panel of genomes harboring the anthrax toxin genes as detailed in Carroll et al. [36]; and (3) the “*B. anthracis*” panel with genomes representative of the different clades of the lineage; Table S3: Public genomes out of the *B. anthracis* lineage and genomes from this study exhibiting ≥ 70% of nucleotide identity (% NI) with *B. anthracis* strain Ames Ancestor; Table S4: MALDI-TOF identification of the isolates using two BRUKER commercial databases (BDAL and SR) in comparison with the closest reference species determined with SNP analyses and with in silico DNA/DNA hybridization; Table S5: Taxonomic assignation and screening of virulence factors of genomes from this study using BTyper3, Summary of closest reference species of each studied strain with in silico DNA/DNA hybridization using Type Strain Genome Server, Detection of pesticidal toxins for SIBC34, SIBC39, SIBC40, SIBC49, SIBC51B and SIBC63 using BtToxin_Digger.
