# Supplementary figures and images for: Get to Know Your Neighbors: Characterization of Close Bacillus anthracis Isolates and Toxin Profile Diversity in the Bacillus cereus Group
Source: Microorganisms. 2023 Nov 7;11(11):2721. doi: 10.3390/microorganisms11112721 (PMC10673079; doi:10.3390/microorganisms11112721)

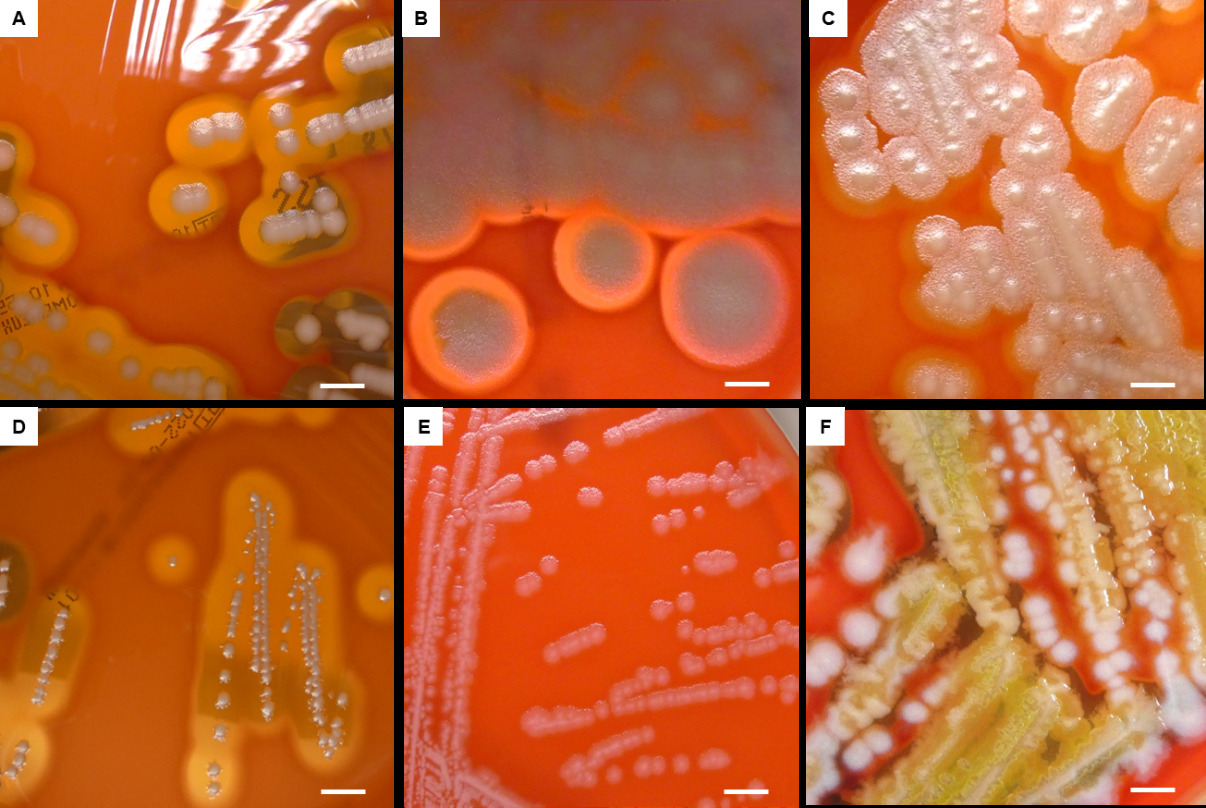

Supplement: Supplementary file 1 [file microorganisms-11-02721-s001.zip › supplementary_data/Figure_S1.jpg]
